# Barriers to and enablers of the promotion of patient and family participation in primary healthcare nursing in Brazil, Germany and Spain: A qualitative study

**DOI:** 10.1111/hex.13843

**Published:** 2023-08-10

**Authors:** Marcus Heumann, Gundula Röhnsch, Edurne Zabaleta‐del‐Olmo, Beatriz Rosana Gonçalves de Oliveira Toso, Ligia Giovanella, Kerstin Hämel

**Affiliations:** ^1^ Department of Health Services Research and Nursing Science, School of Public Health Bielefeld University Bielefeld Germany; ^2^ Division Qualitative Social and Education Research, Department of Education and Psychology Freie Universität Berlin Berlin Germany; ^3^ Fundació Institut Universitari per a la Recerca a l'Atenció Primària de Salut Jordi Gol I Gurina (IDIAPJGol) Barcelona Spain; ^4^ Nursing Department University of Girona Girona Spain; ^5^ Primary Care Directorate, Barcelona Regional Management Institut Català de la Salut Barcelona Spain; ^6^ Center of Biological and Health Sciences Western Paraná State University—UNIOESTE Cascavel Brazil; ^7^ Department for Health Administration and Planning, National School of Public Health Fundação Oswaldo Cruz Rio de Janeiro Brazil

**Keywords:** chronic disease, family caregiver, patient education as topic, patient participation, person‐centred care, primary health care, qualitative research

## Abstract

**Background:**

Most health systems are insufficiently prepared to promote the participation of chronically ill patients in their care. Strong primary health care (PHC) strengthens patients' resources and thus promotes their participation. The tasks of providing continuous care to people with chronic diseases and promoting self‐management are the responsibility of PHC nurses. Recent research assessing enablers of or barriers to nurses' efforts to support patients' participation has mostly not considered the special situation of patients with chronic diseases or focused on the PHC setting.

**Objective:**

To investigate enablers of and barriers to PHC nurses' efforts to promote the participation of chronically ill patients in their care.

**Methods:**

We interviewed 34 practicing PHC nurses and 23 key informants with advanced knowledge of PHC nursing practice in Brazil, Germany and Spain. The data was analyzed using thematic coding.

**Results:**

We identified four categories of barriers and enablers. (1) Establishing bonds with patients: Interviewees emphasized that understanding patients' views and behaviours is important for PHC nurses. (2) Cooperation with relatives and families: Good relationships with families are fundamental, however conflicts within families could challenge PHC nurses efforts to strengthen participation. (3) Communication and cooperation within PHC teams: PHC nurses see Cooperative team structures as a potential enabler, while the dominance of a ‘biomedical’ approach to patient care is seen as a barrier. (4) Work environment: Interviewees agreed that increased workload is a barrier to patient participation.

**Discussion and Conclusions:**

Supporting patient participation should be acknowledged as an important responsibility for nurses by general practitioners and PHC planners. PHC nurses should be trained in communicative competence when discussing participation with chronically ill patients. Interprofessional education could strengthen other professionals' understanding of patient participation as a nursing task.

**Patient or Public Contribution:**

This study is part of a research project associated with the research network ‘forges: User‐oriented care: Promotion of health in the context of chronic diseases and care dependency’. The study's focus and provisional results were discussed continuously with partners in health and social care practice and presented to and discussed with the public at two conferences in which patient representatives, professionals and researchers participated.

## 1 INTRODUCTION

Actively making complex care decisions and taking part in work, family and social life simultaneously can be overwhelming for chronically ill patients and their families. Such efforts are associated with high levels of psychosocial stress.^1^, ^2^, ^3^ To maintain their participation in health care and social life, affected persons need support from health professionals.^4^, ^5^, ^6^ Successful patient participation in health care is achieved when patients and health professionals cooperate in care as coproducers. Patient participation in the context of chronic diseases is understood in this context as (a) participation in health care with respect to patients' involvement in care decisions and ability to adopt an active role in self‐care and self‐management and (b) patients' ability to continue participating in their lifeworlds despite their chronic illnesses.^7^

Healthcare systems struggle to address patient participation sufficiently. Most such systems are designed to treat acute health problems but are insufficiently prepared to promote patients' psychosocial health or participation.^8^ Strong primary health care (PHC) is considered important for providing comprehensive, continuous care for persons with chronic diseases over different courses of illness. Evidence has shown that it is capable of strengthening chronically ill patients' resources and contributes to slowing the progression of the disease as much as possible.^9^, ^10^, ^11^ One of the tasks of PHC professionals is to enable chronically ill people to lead a life that is as normal as possible and to support their social participation and involvement in their own care.^12^, ^13^, ^14^ In many countries, PHC nurses assume particular responsibility for providing ongoing care for people with chronic conditions and carry out various tasks to support their participation.^15^, ^16^, ^17^, ^18^ For example, PHC nurses promote self‐management activities for patients with chronic diseases and their families during home visits. Additionally, they advise and accompany patients and their families in aspects concerning the organization of care and everyday life with chronic diseases.^19^, ^20^ In recent decades, PHC nurses have become increasingly responsible for monitoring the course of chronic diseases and their symptoms. Sometimes, they assume extended clinical tasks, such as administrating medicine, as part of advanced nursing practice roles.^16^, ^21^, ^22^, ^23^

A concept analysis conducted by Sahlsten et al.^24^ ^,p.6^ outlines four characteristics of the concept of patient participation in nursing care: An (1) established relationship, (2) the surrender of some power or control by the nurse, (3) shared information and knowledge and (4) active mutual engagement in intellectual and/or physical activities. Multiple studies have explored the potential enablers of and barriers to nurses' efforts to support patient participation in terms of the following characteristics:

An *established relationship* has been identified as a key caring concept by a concept analysis of patient participation.^25^ Heumann et al.^26^ state that collaborative relationships between PHC nurses and patients must be built on mutual trust.

*Power and control* have been viewed by some scholars as a condition for patient participation.^27^, ^28^ Kao et al.^29^ claimed that power imbalances are enhanced by an authoritative culture in healthcare practice. They argued that such imbalances can be a barrier to patient participation.

The *information that nurses provide to patients* could influence the patients' ability to participate, for example, in care decisions.^25^ Tobiano et al.^30^ emphasized the fact that nurses rely on information from patients to provide targeted care. The studies analyzed by Angel and Fredriksen^28^ indicated that the amount of information a nurse shares with a patient could be influenced by their relationship.

The *willingness and ability of nurses and patients to engage in activities* designed to involve patients and families are potential conditions for patient participation.^26^, ^28^ Patients' ability to engage in activities despite their illness and nurses' competence to motivate patients to take a more active role in their health care are considered relevant in this context.^28^, ^30^, ^31^ However, particularly vulnerable patients whose abilities are under a great deal of pressure from a chronic disease might be unable or unwilling to engage in participatory activities.^7^, ^32^

Increased participation has the potential to promote quality of care and invigorated self‐efficacy for patients.^33^ However, previous studies have shown that ensuring patient participation in PHC nurses' daily practice can be challenging. Therefore, more research on the conditions that can enable patient participation and the barriers that currently hinder it remains necessary. Previous research has rarely addressed the enablers of and barriers to patient participation explicitly. These facts have rather been indicated by the results of studies focusing primarily on the organization and procedures involved in participation processes between patients and nurses. In addition, most studies have not taken into account the special situation of patients with chronic diseases or the views of nurses as their main point of contact in PHC. Our study addresses this issue by conducting an in‐depth analysis of enablers of and barriers to PHC nurses' efforts to promote the participation of chronically ill patients in their care. To strengthen the transferability of our results, we integrated nurses' experiences in PHC in three healthcare systems: Brazil, Germany and Spain.

## 2 METHODS

### 2.1 Aim and research questions

The aim of this study was to investigate enablers of and barriers to PHC nurses' efforts to promote the participation of chronically ill patients In their care. To achieve this goal, the following research questions guided our analysis:

1. Which interactional and structural aspects enable or hinder nurses' ability to promote the participation of chronically ill patients in their care?
2. To what extent do PHC nurses reflect on their own role in the promotion of patient participation and the way they shape it, and to what extent do PHC nurses experience practical constraints on this role?

The study is reported according to the Consolidated Criteria for Reporting Qualitative Research.^34^

### 2.2 Study design

For our study, we chose a qualitative design. Data was collected using expert interviews, a special form of semistructured interviews that addresses interviewees' practical and context knowledge.^35^, ^36^ We interviewed practicing nurses (PNs) and key informants (KIs) as experts with practical and context knowledge of PHC nursing practice.^36^ The interviews addressed the interviewees' subjective experiences and perceptions of PHC nurses' role in supporting chronically ill patients' participation. Data were analyzed using thematic coding following Flick.^37^

### 2.3 Study context

This qualitative study is a substudy of a larger research project that aims to identify the options and conditions associated with the implementation of user‐oriented care approaches in primary and long‐term care with a particular focus on the role and tasks of nurses in Brazil, Germany and Spain.^12^ The selection of the three countries took into account different healthcare systems and different stages in the conceptual development of user‐oriented care approaches. In addition, the three countries represent different ranges of responsibility for PHC nurses.^12^

### 2.4 Sample

We used purposive sampling. Participants who were consulted as KIs were researchers in the field of PHC nursing or PHC coordinators at the regional or national level. PHC nurses were at least educated as registered nurses and worked in PHC nursing. We took into account health system‐specific circumstances. In Brazil, we, therefore, included only PHC nurses who worked in the family health strategy, while in Spain, we limited the sample to PHC nurses who worked in PHC centres (Contros de Salud). Nurses in Germany are rarely integrated into PHC facilities. They partly assume tasks within the scope of PHC in the context of home care services, which, however, focus mainly on long‐term care and are institutionally separated from the practice of general practitioners (GPs).^38^, ^39^ PHC nursing is associated mostly with model projects. Therefore, we specifically interviewed nurses in Germany who worked on such model projects. More information about the sample is provided elsewhere.^12^ We interviewed PNs and KIs in four states in Brazil, seven states in Germany and four autonomous communities (regional states) in Spain. Throughout this process, we considered the diversity of and regional differences across the three countries.^12^ Sampling continued during the data collection process until data saturation was reached and no significant additional information was expected from additional interviews.

We initially aimed to interview 15–20 persons per country, half of whom would be practicing PHC nurses, while the other half would consist of KIs. In fact, we interviewed 23 KIs and 34 practising nurses in total (see Table 1). None of the interviewees were interviewed repeatedly. Most KIs were originally trained as nurses. Only a few interviewees (*n* = 3) had other types of professional education (see Table 1). Participants in this study were recruited with the support of local professional organisations and cooperation partners in the three countries. Some interviewees were also recruited through previously interviewed persons. Details about the recruiting process are provided in another publication.^12^

**Table 1:** Sample.

Participant characteristics	Total	Brazil	Germany	Spain
Participants	57	20	18	19
Key informants	23	10	6	7
Researchers	9	5	2	2
Coordinators/PHC managers	14	5	4	5
Practicing PHC nurses	34	10	12	12
Sex				
Male	8	0	4	5
Female	49	20	14	14
Professional background				
Nurse	54	20	16	18
Physician	2	0	1	1
Economist	1	0	1	0

Abbreviation: PHC, primary health care.

### 2.5 Data collection

We used an interview guide that was discussed among M. H., G. R. and K. H. until consensus was achieved. The construction of the interview guide was based on the episodic interview approach. Accordingly, it consisted of targeted interview questions and questions that encouraged interviewees to narrate concrete situations.^37^, ^40^ The interview guide was structured around four themes: (a) the tasks of family and community nurses and task division and collaboration with physicians (and other health professionals) in chronic care; (b) nurses' responsibility for individual participation in the care of people with chronic diseases and their families; (c) nurses' responsibility for the promotion of participation in groups and the participation of communities and (d) an overall assessment of the relevance and facilitating and hindering conditions of the promotion of patient participation by nurses. The interview guide was developed in German and later translated into English, Portuguese and Spanish. The interview guide was pilot tested in all three countries. Only small modifications were necessary after pilot testing; hence, all three pilot interviews were included in the data analysis. The interview guide can be found in Supporting information: Material.

The interviews were conducted between August 2019 and December 2020; some of the interviews were conducted face‐to‐face, while others were conducted online (due to the COVID‐19 pandemic). The face‐to‐face interviews were conducted either in the working environment of the interviewees or in quiet rooms in the researcher's working environment. Either one of the authors or a set of interviewers (Brazil: two female nursing students and one female PhD student; Germany: one PhD student; Spain: one PhD student and one male postdoctoral researcher) who were trained by one of the authors and had advanced language skills in the respective languages conducted the interviews.

Other than the interviewers, authors and interviewees, no other persons were present during the interviews. The interviewees and interviewers in Germany and Spain did not have a relationship before the interviews. In Brazil, in seven cases, the interviewers and interviewees were known to each other from a private context (*n* = 2) or through university teaching (*n* = 5). In all other cases, interviewers introduced themselves before the start of the interview and explained the aim of the study. Field notes were not systematically prepared and analyzed. The lengths of the interviews varied between approximately 21 and 133 min. The average length of the interviews was 59:39 min.

### 2.6 Ethics and data protection

The study was approved by the Bielefeld University Ethics Committee (No. 2018‐170) following the ethical principles of the German Society for Psychology. All participants were informed about the aims of the study, the procedure of the interview and their right to withdraw their consent to participate in the study using a participant information sheet. They were also informed about the usage and processing of the collected data as well as the data protection measures being employed. All participants gave oral and written consent to participate in the study after being given the chance to read the participant information sheet and ask further questions. After agreeing to participate in the study, none of the potential interviewees dropped out. All interviews were audio recorded after obtaining the interviewees' consent.

### 2.7 Transcription and data analysis

All interviews were first transcribed verbatim. The interviews in Spanish and Portuguese were translated into English and analyzed based on the translated transcripts. The interviews in German were analyzed based on the original transcripts. All persons who performed the translations into English had advanced knowledge of the original language (Portuguese/Spanish) as well as English. To ensure the trustworthiness of the data, the translations were subsequently checked by at least one co‐author.^12^ The transcripts were anonymized by removing personal information and other details that could identify the interviewees (as well as location details).

We used VERBI GmbH MAXQDA software (Version 2020) to analyze the data. In the first step, we structured the material obtained from the interviews. Therefore, we coded all interviews by allocating the data to deductively developed themes that were deduced from the themes contained in the interview guide.^37^ In the second step, the two categories ‘Facilitating and hindering aspects of patient participation’ and ‘Conditions for nurses when supporting patient participation’ were selected to understand the interviewees' perception of aspects that hinder or promote the participation of chronically ill patients in their care. We applied the three research questions to the data and assigned them to inductively developed thematic areas. M. H., G. R. and K. H. discussed the categorization to those thematic areas until they reached consensus. The discussion resulted in the following categories: ‘Establishing bonds with patients’, ‘Cooperation with relatives and family’, ‘Communication and cooperation within primary health care teams’ and ‘PHC working environment’. In a third step, we inductively deduced subcategories from the interview material to differentiate among the categories more thoroughly. Our categories were based on our theoretical preunderstanding of concepts and theories related to the role of nurses in promoting participation and self‐management in the context of chronic diseases.^12^

### 2.8 Reflexivity and rigour

To ensure rigour, we used data and investigator triangulation.^41^, ^42^ Data from three different countries were triangulated. Within the countries, we interviewed KIs and PNs from different regions. The interviewed nurses practiced in both rural and urban areas. They were qualified as registered nurses. Some of them possessed degrees at the master's and PhD levels. Investigator triangulation was ensured by involving authors from all the countries involved as well as different professional backgrounds at all stages of the research process. The authors' professional backgrounds are in nursing (E. Z., B. T.), medicine (L. G.), social sciences (G. R., K. H.) and public health (M. H.). Despite the fact that the research fields of all authors are in areas related to public health, the authors are able to contribute different perspectives and research experiences in different subdisciplines of public health and nursing science. All authors have expertise in qualitative research. The first author is a PhD student, while all other authors are senior researchers with high levels of expertise in qualitative research methods.

## 3 FINDINGS

In the interviews, the nurses and KIs primarily referred to enablers of and barriers to their efforts to promote patient participation that originate in nurses' interactions with patients (Section 3.1) and their relatives or families (Section 3.2). They also repeatedly reflected on structural enablers and barriers related to their work with other professions or in PHC teams (Section 3.3). Furthermore, they identified structural conditions of their working environment that enabled or hindered their efforts to support the participation of chronically ill patients (Section 3.4). Table 2 describes the categories and provides exemplary quotations for the respective categories.

**Table 2:** Categories, definitions and example quotations.

Category	Definition	Example quotations^a^, ^b^
Establishing bonds with patients	Statements referring to bonds that PHC nurses develop with patients and that are perceived as hindering or enabling with regard to the participation of chronically people.	Therefore, I realize that there was an advance in the sense of having a greater bond with this patient, being more aware of where this patient lives, the context of this user, right? This does interfere with the way we will guide, the way we will treat this patient. (Br KI E9, 22)
		That you are simply not this hierarchically dominant person but rather that you try to find yourself on the same wavelength and on the same level with the patient. (Ge PN E16, 70)
		Above all, [it is important] to promote a therapeutic relationship. That is to say, that there is a relationship of trust so that there is adherence to treatment. You have to have a relationship of trust towards us. If patients do not trust us, ‘I can tell them what I want, that, when I walk out the door, that patient will ignore me’. (Sp PN E11, 41)
Cooperation with relatives and families	Statements referring to PHC nurses' engagement with chronically ill patients' families as a condition that enables or hinders patient participation.	Sometimes, we expect something from the family, but there are other things that are happening in the inner‐circle of this family that we don't see, and if we can't see it, we won't understand why the family is sometimes not so participative (Br KI E4, 82)
		So, you really have to differentiate when it makes sense and is necessary to involve the family member; for example, it could be necessary, when it is about someone who is changing their diet, who wants to work on their eating habits, and you have, for example, a family system, and there is the mother, who has just been diagnosed with diabetes, [and she] is suddenly supposed to change her diet, and then the family system might say, yes, the mother can change her diet, but that should not mean that the others also change their diet, or the others support her in doing so. (Ge KI E4, 17)
		But when you really see family dynamics, and when you can involve the family in the care (with the patient), it really is at home. Because you see the one who comes to visit and is really there. How are household chores distributed? Who will buy? Who will run the washing machine? (Sp PN E5, 73)
Communication and cooperation within PHC teams	Statements referring to working relationships with nurses and other professions in PHC teams (especially GPs) that nurses view as enabling or hindering with regard to patient participation and the role that PHC nurses play in PHC teams.	From the physician, they understand, of course, all the importance of doing the stratification, but it's a difficulty that we have. If we had their collaboration, I think we would advance much more, and it would be easier on us. They have difficulty doing the follow‐up. For them, they see the patient, make the consultation, prescribe, send home. (Br PN E11, 69)
		So, I think that what colleagues actually report from practice, when it comes to negative examples, to a large extent, there are coordination problems in the sense of communication, that the doctor, for example, if we stay with the topic of dementia and show the possibilities of nursing care, for example, then the information that the GP gives partly does not correspond to what the nursing professionals would advise or recommend. (Ge KI E3, 9)
		In other words, there are medical groups or physicians from an individual point of view that are not used to the nurse evaluating the patient, and sometimes they see it as a loss for their patient. When you have to assess whether that represents an improvement for the patient, we have to join forces and work for the best for the patient. (Sp KI E8, 58)
PHC working environment	Statements referring to work environments that PHC nurses view as enabling or hindering with regard to their efforts to support the participation of chronically ill patients.	I have a place for nursing consultations, but it is only one. And there are the residency students and the doctor intern group who also use it. We have to schedule to use it because there aren't enough places. Sometimes, I even tell the users that I have a lot of people to work; however, I do not have enough space. (Br PN E16, 53)
		Now, I'll say that I don't have mobile reception in the villages to turn on my laptop; that just makes it more difficult for me then because I lose time. If I really want to do certain things on the spot, I simply can't do it because I don't have mobile reception and my SIM card in my laptop doesn't work. (Ge PN E6, 247)
		It is important that we have that autonomy and that support. The negative things: that they see nurses as people who do not work. I mean: the work of the nurse, many times, is seen as something technical. As if it were the fact of ‘following a protocol’, ‘doing a certain technique’ (…) But there are some nurses who do not think that health education is part of your daily work. (Sp PN E14, 123)

Abbreviations: GP, general practitioner; KI, key informant; PHC, primary health care; PN, practicing nurse.

^a^ Quotations were translated from the original languages into English and edited for grammar to improve their readability.

^b^ The information provided in brackets following the direct quotations refers to the country in which the quoted person worked (Br = Brazil, Ge = Germany, Sp = Spain) and to whether the person was a PN or a KI.

### 3.1 Establishing bonds with patients

From the viewpoint of the interviewees, PHC nurses could strengthen chronically ill patients' participation in their care, especially if they established bonds with their patients: ‘The bond, it is extremely important for the patient, for the user to comply to our orientation’ (Br PN E8, 92) (the information provided in brackets following direct quotations refers to the country in which the quoted person worked (Br = Brazil, Ge = Germany, Sp = Spain) and to whether the person was a PN or a KI). For this purpose, nurses must first ‘become involved’ with the patient. This specifically means they should get to know the patients' views and behaviours and ‘have a better overview of what the [patients'] needs are in the first place and also to get a feeling of what could benefit the patient’ (Ge KI E3, 13). Home visits were an important tool for nurses to obtain a more comprehensive overview of the patients' life situation.

The interviewees found it crucial for nurses to know the ‘art of listening’ to understand the subjective reality of the patients' lives:

What helps is to listen to them [the patients] and from here, to know how to understand and that they are feeling understood and that they feel that you understand what is happening to them. (Sp PN E6, 71). (Quotations were translated from the original language to English and grammatically edited to improve readability.)

An understanding of their patients' living situations helps PHC nurses establish ‘therapeutic pacts’ (Sp PN E11, 42) with them, for example, to collaboratively determine how patients can adopt healthier diets. However, the interviewees also pointed out that building bonds to support long‐term participation and more personal responsibility for patients can be energy‐ and time‐consuming for nurses. This is especially true because patient's interest in and possibilities of actively engaging in their own health and care vary widely. For poorer patients, for example, fulfilling existential needs such as being able to buy food can be such a great challenge that they hardly have the resources to deal with their health concerns in the long term. From the interviewees' point of view, it is primarily important for those patients to cope with acute health problems as rapidly as possible, for example, to be able to work regularly and earn a living. These patients often do not have time for continuous contact with PHC nurses to develop possible long‐term strategies with them. The interviewees also perceived that older patients can become overwhelmed by a multitude of support and help offers and sometimes perceive self‐management support as a burden:

Then, they [the patients] also say, ‘Oh, the physiotherapist already comes twice a week and the home care service and so on and then someone takes me for a walk, so actually I don't want that and actually it's too exhausting for me too’. (Ge PN E16, 72)

Insecure patients might rely on nurses' recommendations so completely that they will rarely take the initiative. Nurses were challenged by introducing patients to more active participation without making them feel that they were on their own. However, some experts critically reflected that nurses do not always allow patients enough space to ‘grow into’ a more active role in care. Simultaneously, PHC nurses sometimes found it tiring to encourage patients' self‐care continuously if the patients were apparently not motivated to engage in this practice: ‘He doesn't move, he doesn't move and of course you reach your limits, you can always talk about it’ (Ge PN E6, 203). In this context, nurses expressed their frustration with patients who did not follow their recommendations and suggestions.

### 3.2 Cooperation with relatives and families

To enable patients to deal with chronic illnesses as autonomously as possible, the interviewed experts found it particularly important for PHC nurses to ‘make an alliance with the family’ (Br PN E5, 153). The nurses saw this not only as beneficial for the patients but also as a chance to promote health consciousness and health‐promoting behaviour in the everyday life of the whole family: ‘We have to talk to the family because it is a chance for the whole family almost at the [same] time’ (Br PN E5, 153). However, it was important for PHC nurses to express appreciation of family members in their role as caregivers: ‘I see the caregiver as a gift’ (Sp PN E6, 63). They emphasized that they had to give attention to the family's situation and that they needed to be there for the relatives to strengthen the existing resources in the family environment. Especially when patients (e.g., due to dementia) had limited cognitive abilities and could no longer make autonomous care decisions or manage their own care, nurses viewed cooperation with families as essential to patient participation. They found it important to address relatives' problems and encourage them to accept ‘that their role is feasible’ (Sp PN E16, 65). The interviewees commented that the participation of families could be promoted if nurses regard them as ‘coproducers’ whose knowledge and skills complement those of the nurses:

I will not make him feel like a person who is there next to me, but … I will devote part of me … [when] I am for one hour – or more or less – at a patient's home, approximately half [of the time] goes to one person and the other half goes to the other. Then, everything we have talked about training and teaching, I explain to him and I explain more things to him. (Sp PN E6, 63)

The interviewed experts found it challenging to deal with conflicts within families. PHC nurses often faced situations where family members disagreed about which care decisions were ‘right’ or ‘best’ for the patient. In these situations, PHC nurses felt that ‘it really goes beyond our means’ (Sp KI E4, 107). Nurses believed that they must maintain a neutral position and show the families that they will not actively intervene in the conflict but rather appreciate every position of the parties involved. However, they always stress the specific needs of the chronically ill person. In this context, the interviewed experts found it important for PHC nurses to have the competence to mediate between family members and the ability to identify, together with all parties, ‘more in‐depth’ underlying problems that could be the reasons for intrafamily conflicts. The nurses emphasized that relatives are often overextended in the care situation, which can lead to tensions within families:

Of course you also notice a lot of tension; the relatives are heavily burdened by the care situation, and sometimes they are very fraught. And yes, sometimes you have to mediate. (Ge PN E7, 39)

### 3.3 Communication and cooperation within PHC teams

The interviewees reported that nurses and physicians work ‘complementarily’ (Sp PN E9, 76). While treating diseases was seen as a medical task, nurses—because they are ‘closer to the patient’ (Br PN E5, 170)—regarded themselves as competent in structuring and organizing participation and health promotion. Despite these different competences and tasks, nurses and physicians should speak ‘the same language’ (Br KI E16, 79). This means, in particular, that care providers in the two professions should not make contradictory recommendations to patients (e.g., in relation to therapy planning). PHC nurses in all three countries observed an overemphasis on the ‘biomedical model’ (Sp PN E6, 6), which they attributed primarily to physicians. PHC nurses found that physicians tended to focus on biomedical aspects of self‐management. From the nurses' perspective, self‐management in the patients' social lifeworld context should be equally strengthened. They felt that this one‐sided perspective limits their ability to increase patients' participation through psychosocial counselling and self‐management support.

Specifically, nurses in Germany expressed that they find it difficult to apply their competences in a strongly hierarchical working relationship. They commented that they have problems promoting patient participation when physicians do not support them in this area:

And when the physician, for example, goes to these people, and one says, ‘I have dizziness’. And then the physician says, ‘Yes, that's just the way it is, you just have dizziness now’. And then the people ask, ‘Can I do something?’, and he says, ‘No, you can't do anything’, then it's very, very difficult for me to go there and then try to explain self‐management or some exercises to them after the ‘god in white’ has said that you can't do anything before. (Ge PN E16, 110)

Where nurses and GPs work in fixed team structures (Brazil and Spain), nurses stressed that it was important for physicians to understand the competences of nurses in involving patients and their relatives in care: ‘You also have to teach the doctors with whom you work that a nurse is more than just someone giving injections’ (Sp PN E15, 21).

The interviewees also stressed that nurses should ‘exercise self‐criticism’ (Sp KI E5, 128) regarding their own competences in health promotion and supporting participation. Nurses considered themselves responsible for continuous reflection on whether they or other team members (especially physicians) are able to allow for patient participation in the care process. They pointed out that overcoming the paternalistic tendencies that have long shaped the relationship between health professionals and patients is challenging:

Many times, nurses were trained in a very authoritarian model to act in health care. So ‘they say: you must do this, you must drink water, eat well, do that, etc’. A very vertical relationship of prescribing care. (Br KI E4, 92)

### 3.4 PHC working environment

According to the interviewees, nurses need a working environment that allows them to have trusting conversations with patients and carry out health promotion and self‐management activities to support patients' participation. This means they require sufficient and appropriately equipped rooms where they can converse with patients about health‐related and sometimes intimate topics without being disturbed. However, some nurses, especially in Brazil, complained that these basic requirements are often not available: ‘The woman feels more at ease when she can undress in a bathroom, or when she has, at least, a folding screen for her to be a little more protected’ (Br PN E8 121). The interviewees noted that a lack of regional infrastructure could be a limiting factor for enhancing patient participation in addition to space and equipment limitations. For example, nurses in Germany reported that insufficient (telephone or internet) network coverage, especially in rural areas, stands in the way of digital conversations with patients: ‘I don't have connection here with my electronic patient record, and then you actually want me to work with Skype here – forget it’ (Ge PN E15, 64).

In contrast to the experts from Brazil and Germany, those from Spain complained less about the infrastructural‐technical working environment. Rather, they perceived the protocols and guidelines according to which they worked as a barrier when they tried to strengthen the participation of particularly vulnerable or hard‐to‐reach patients. They criticized the fact that these guidelines do not offer the flexibility they need to consider the complex problems of these groups sufficiently: ‘But protocols have not always been developed that allow [us] to reach certain groups that have greater difficulty in implementing self‐care’ (Sp PN E12, 49).

The interviewed experts from all three countries regarded increasing workload as a barrier to supporting patients' participation. They particularly wished for ‘more time to see patients’ (Sp PN E3, 92) so that they can engage with their individual situations and build relationships of trust. The interviewees' statements reflect that all three countries have implemented initiatives to strengthen patient participation and health promotion as a nursing task. The interviewed PHC nurses, however, still complained that in practice, other particularly labour‐ and time‐intensive tasks are prioritized. The tasks that are considered especially time‐consuming varied among the countries. In Spain, the nurses mentioned clinical tasks in particular: ‘Certain patients are occupying me through half of the morning, so that they come to measure their blood pressure’ (Sp PN E6, 119). Nurses in Brazil referred more specifically to administrative and management activities: ‘We also manage the entire staff’ (Br KI E11, 141). In Germany, the interviewees across the different model projects noted that payments for nurses' work towards promoting self‐management and participation are not regularly covered in the German healthcare system; therefore, such tasks are usually achieved more as a byproduct of their ordinary work: ‘You have the difficulty that they first of all need a financing basis or a contractual basis in order to be able to provide the service that we provide’ (Ge KI E13, 98).

## 4 DISCUSSION

The aim of this study was to investigate the enablers of and barriers to PHC nurses' efforts to promote the participation of chronically ill patients in their own care. Our analysis identified four categories related to conditions that hinder or facilitate nurses' efforts to strengthen the participation of patients with chronic diseases. Two categories refer to the interactions between PHC nurses and patients and their families, respectively, that is, (1) establishing bonds with patients and (2) cooperation with relatives and families. The other two categories pertain to structural aspects, that is, (3) communication and cooperation within PHC teams and (4) the PHC working environment.

We identified ‘establishing bonds with patients’ and ‘cooperation with relatives and families’ as categories that pertain to the conditions that hinder or facilitate PHC nurses' efforts to strengthen chronically ill patients' participation at the interactional level. Our results correspond to other research that has emphasized the relevance of relationship‐building between nurses, patients and their families as a condition for patient participation. Sahlsten et al.^24^ and Nilsson et al.^25^ highlighted this aspect in their concept analyses. While other studies have emphasized the fact that power imbalances shape the relationships among nurses, patients and their families,^24^, ^28^ our study results emphasize the importance of understanding the patients' lifeworld and the families' individual situation when supporting patient participation. This approach is related to the concept of ‘lifeworld‐led care’;^43^ in this context, Dahlberg et al.^44^ highlighted the fact that ‘lifeworld knowledge’ is crucial for what they consider good nursing. Lifeworld knowledge corresponds with ‘shared information and knowledge’, which was emphasized as a characteristic of patient participation by Sahlsten et al.^24^ Additionally, our study results refer to another characteristic of patient participation: active engagement in activities.^24^ We found that this aspect is relevant not only for the individual patient's participation but also for the involvement of chronically ill patients' families. In line with other studies, our results emphasize the relevance of family involvement as an essential element of nursing care.^45^, ^46^, ^47^ In this context, our results highlight the fact that nurses' ability to support families when playing a more active role in caring is viewed as a condition for patient participation. In line with Doekhie et al.,^48^ however, our study notes that the involvement of family caregivers is experienced as potentially challenging for patient participation when family members do not cooperate with PHC professionals to promote stronger patient participation.

Structural factors that represent conditions for promoting patient participation have been underrepresented in previous research. Our study expands on this topic by addressing the categories ‘Communication and cooperation within PHC teams’ and ‘PHC working environment’. Our results suggest that patient participation can be facilitated if PHC nurses and GPs complement each other and if they align the information they provide to the patient. Correspondingly, the results of a study conducted by Carvalho et al.^49^ indicated that PHC professionals consider discussions and exchanges within PHC teams to be an important enabler when trying to encourage patient participation. However, more research addressing PHC team‐related enablers and barriers to patient participation remains necessary. Our study participants noted that negative work environment conditions such as increasing workloads are an important barrier to nurses' attempts to support patient participation. Previous research has affirmed that conditions in the work environment can enable or hinder patient participation.^26^ Multiple studies have indicated that PHC nurses and other PHC professionals experience increasing workloads in their daily practice^50^, ^51^, ^52^, ^53^; the impact of this issue on patient participation, however, remains underresearched. Apart from workload, our results suggest that poor PHC facilities and regional infrastructure (e.g., inadequate telephone or internet network coverage) can be perceived as potential barriers to patient participation. This aspect should be addressed by future research, as should the implications of the inflexible guidelines that define PHC nurses' scope of practice.

### 4.1 Implications for policy and practice

Health professionals, especially nurses, should be supported in their attempts to adopt a more systemic perspective on chronic care that takes into account the lifeworld of both patients and their families as well as their individual challenges and competences. We recommend that patients should be encouraged to play more active roles in conversations with them. Previous research, however, has shown that PHC nurses working in this context should acknowledge chronically ill patients’ vulnerability. Namely, they must accept that due to the severity of some chronic diseases, patients and families might not be able to play a more active role in their own care (any longer).^7^

Our study results show that supporting patient participation must be identified as a relevant task for nurses by the relevant guidelines as well as by PHC management. Accordingly, PHC nurses should be given sufficient time and resources to perform the tasks related to this responsibility, and GPs and other PHC team members must accept participation support as an important part of nursing practice. To support chronically ill patients' participation, PHC teams should establish regular communication structures within the teams that allow them to adopt a cooperative care approach. PHC nurses must have the autonomy and flexibility they need to adjust their care approach to individual patients' and families' needs.

Strengthening nurses' competences with regard to patient participation should be considered in PHC nurses' training and continuous education. This issue particularly encompasses communicative competences in conversations with patients, their families and other health professionals. For example, PHC nurses should be trained to mediate when they witness conflicts within families and to emphasize the patients' interests. When cooperating with other health professionals, PHC nurses must be educated to raise other health professionals’ awareness of the special needs of chronically ill people. Interprofessional education could strengthen the mutual understanding of tasks and responsibilities among nurses, GPs and other health professionals and could help them develop aligned communication strategies.

### 4.2 Strengths and limitations

During the study process, we used several measures to ensure trustworthiness of the data analysis.^54^ We kept detailed records of our data and the steps involved in the data analysis process. Additionally, we used data and investigator triangulation^42^ at all stages of the research process and discussed the interview guide and the analyzed categories among the authors until consensus was reached. This sample takes into account different PHC models and nursing roles as well as regional differences across the countries. The variety of the sample allowed us to analyze enablers and barriers detached from a single, country‐specific PHC model or PHC nursing role. This aspect is the major strength of our study.

However, some methodological aspects of our study must be considered limitations. First, the study did not include interviews with patients or the family members of chronically ill people. Research has indicated that patients and nurses may understand patient participation differently.^55^ Future research should therefore consider patients' and families’ views of barriers to and enablers of patient participation in PHC nursing. Additionally, due to limited resources and availability of the participants, data analysis results were not discussed with the participants (using, e.g., member checks). This lack could limit the transferability of the results. Another potential risk for bias arises from the fact that the interviews were conducted in different languages due to the need to translate the transcripts.^56^ To limit this risk of bias, the authors educated the interviewees and translators and carefully reviewed the translations.

## 5 CONCLUSION

Barriers of and enablers to nurses' efforts to support the participation of chronically ill patients can emerge from the interaction with patients and families as well as from structural conditions referring to the cooperation and communication that occurs within PHC teams and in the PHC working environment. To support patient participation, nurses must have the resources and competences that can allow them to develop close relationships with patients and families and to view them in the context of their living situation. The ability to accomplish this task can be challenged by team structures and unfavourable work environment conditions such as heavy workloads and time‐consuming routine tasks. Therefore, participation support must be acknowledged as an important task for PHC nurses by the relevant guidelines, PHC management, GPs and other professionals.

## AUTHORS CONTRIBUTIONS

Marcus Heumann, Gundula Röhnsch and Kerstin Hämel conceptualized the study and developed the interview guide. All authors recruited and contacted potential interview partners and were involved in data collection. Marcus Heumann, Gundula Röhnsch and Kerstin Hämel analyzed the data in the first step. Marcus Heumann wrote the first draft of the manuscript. Gundula Röhnsch and Kerstin Hämel reviewed the first draft and were major contributors to the further interpretation of data and writing of the manuscript. All authors commented and revised the manuscript multiple times. All authors have approved the final manuscript and agree to be accountable for all aspects of the work in ensuring that questions related to the accuracy or integrity of any part of the work are appropriately investigated and resolved.

## CONFLICT OF INTEREST STATEMENT

The authors declare no conflict of interest.

## ETHICS STATEMENT

The study was approved by the Ethics Committee of Bielefeld University (No. 2018‐170). The assessment was carried out in accordance with the ethics guidelines of the Deutsche Gesellschaft für Psychologie e. V. (German Psychological Society). Informed consent was obtained from all study participants.

## Supporting information

Supporting information.

## Data Availability

Given the potentially disclosive nature of entire interview transcripts, they will not be made freely publicly available. They will be deposited at Bielefeld University and are available from the corresponding author upon reasonable request.
