# Supplementary material for: Barriers to and enablers of the promotion of patient and family participation in primary healthcare nursing in Brazil, Germany and Spain: A qualitative study
Source: Health Expect. 2023 Aug 10;26(6):2396–408. doi: 10.1111/hex.13843 (PMC10632623; doi:10.1111/hex.13843)
Supplement: Supplementary file 1 — Supporting information. [file HEX-26--s001.pdf]

Guide for interviews with practicing PHC-nurses and key informants

In our study we analyze how family and community nurses (Sp)/nurses of the family health strategy (Br) involve patients/users in their care. We conduct this study in Germany, Spain and Brazil.

More specifically, we are interested in how do nurses (a) work with patients and their families individually, and (b) how they work with groups and communities in order to promote health and to address the needs of people with chronic diseases.

We will interview several experts in primary health care and nursing, like directors or coordinators in primary health care and health centers. And of course, we interview nurses who work in direct patient care.

I will ask you several questions, for example on your tasks in certain areas and how you involve users in their care. If I ask questions that you are not able to or you do not want to answer, please let me know. I cannot always estimate how familiar the different interview partners are with the topics. We are asking the same questions in all of the three countries; however, we know that nurses in different countries have different profiles and because of that can tell us more or less according to the different questions.

Introduction

1. *Key informants & Practicing nurses:*
   At the beginning could you please introduce yourself? What is your education? What is your occupational position and what are your current tasks.

Tasks of family and community nurses; task division and collaboration with doctors (other health professionals) in chronic care

2. Could you give me an overview on the main tasks of the nurses in in your facility (Ger)?/ Your health centre(s) (Esp)/In your familiy health team(s) (Bra)
   a) *Key informants:*
      Could you tell me more in detail? Which tasks do the nurses in your facility/health centre(s)/your family health team(s) assume in the area of health promotion? Could you describe a – in your view – typical nursing activity in health promotion, that illustrates how the nurses act?
      Practicing nurses:
      Could you tell me more in detail? Which tasks do you assume in the area of health promotion? Could you describe a – in your view – typical activity in health promotion, that illustrates how you act?

   b) *Key informants:*
      Could you tell me more in detail: Which tasks do the nurses assume in the care of chronically ill patients/users? Could you tell me a case example, that - in your view – shows which tasks nurses typically assume in the care of chronically ill patients?
      Practicing nurses:
      Could you tell me more in detail: Which tasks do you assume in the care of chronically ill patients/users? Could you tell me a case example of a chronically ill patient that - in your view – shows which tasks you typically perform in the care of chronically ill patients?

c) *Key informants:*
Compared to other health centres, do the nurses in your facility/health centre(s)/family health team(s) here assume special or other tasks in the care for chronically ill patients? What are these tasks?
Practicing nurses:
Compared to your colleagues, do you perform special or other tasks in the care for chronically ill patients? What are these tasks?

d) *Key informants:*
If you look at the last years in your facilities/health centre(s)/familiy health team(s), have there been changes in the activities nurses are doing in the care for chronically ill patients? Could you please describe these changes for me.
Practicing nurses:
If you look at the last years in your health center/family health team, have there been changes in your activities in the care for chronically ill patients? Could you please describe these changes for me.

3. *Key informants:*
In your facilities/health centre(s)/family health team(s), how the division of tasks between the nurses and the primary care doctors in caring for chronically ill people designed? Could you please describe for me how the task division looks like?
Practicing nurses:
In your facilities/health centre(s)/family health team(s), how is the division of tasks between you and the doctor(s) in caring for chronically ill people designed? Could you please describe for me how the task division looks like?

a) *Key informants & practicing nurses: Afterthought:* Could you give me please a "typical" case example for this task division?

b) *Key informants:*
In your opinion, what is going well in the collaboration between doctors and nurses in the care for chronically ill patients? And vice versa: What could be better in this collaboration? (Can you tell me a situation so that I can imagine it better)
Practicing nurses:
In your opinion, what is going well in the collaboration with the doctor(s) in the care for chronically ill patients? And vice versa: What could be better in this collaboration? (Can you tell me a situation so that I can imagine it better)

Individual participation of people with chronic diseases and their families in the care as a responsibility for nurses

Key informants:
Now I would like to focus on the nurses' practices to involve people with chronic diseases and their families in the care.
Practicing nurses:
Now I would like to ask you questions about how you involve people with chronic diseases and their families in their care.

4. *Key informants:*
   How do nurses in your health centre(s)/family health team(s) strengthen the competencies of patients so that they can better deal with their own illness? Can you please tell me about such approaches?
   Practicing nurses:
   How do you strengthen the competencies of your patients so that they can better deal with their own illness? Can you please tell me about such approaches?

5. *Key informants:*
   When you think of the everyday life of chronically ill people: How do nurses make it easier for people to live with their illness in everyday life? Can you give me a case example?
   Practicing nurses:
   When you think of the everyday life of your chronically ill patients/users: How do you make it easier for your patients/clients to live with the illness in everyday life? Can you give me a case example?

6. *Key informants:*
   How/in which way do nurses involve patients in decisions regarding their own care? Could you tell me that by using an example or a situation?
   Practicing nurses:
   How/in which way do you involve patients/users in decisions regarding their own care? Could you tell me that by using an example or a situation?

7. *Key informants:*
   Are there also situations where the "freedom" of patients to co-decide on their care reaches its limits? Can you tell me about such a situation?
   Practicing nurses:
   Are there also situations where the "freedom" of your patients to co-decide on their care reaches its limits? Can you tell me about such a situation?

8. *Key informants:*
   How do nurses cooperate with family members of chronically ill people? Can you tell me a case example? Is there anything that makes it easier or more difficult for nurses to involve family members?
   Practicing nurses:
   How do you cooperate with family members of your chronically ill patients? Can you tell me a case example? Is there anything that makes it easier or more difficult for you to involve family members?

9. *Key informants & practicing nurses:* If you think of different population groups, e.g., different age groups, social classes, people with certain chronic diseases: Do you see any differences in whether and how these different groups can be or want to be involved in their care? Could you give an example?

10. *Key Informants:*
    In your experience, what working conditions in your health centre(s) make it easier for nurses to promote the involvement of users in their care?
    (Afterthought: What are other working conditions that facilitate user participation in their care)
    (Examples: time/workload)
    Practicing nurses:
    In your experience, what working conditions in your facility/health center(s)/family health team

make it easier for you to promote the involvement of your patients in their care?
(Afterthought: What are other working conditions that facilitate user participation in their care)
(Examples: time/workload)

a. *Key informants:*
   And vice versa, which working conditions hamper the promotion of the participation of the users in their care?
   (Afterthought: Can you name further working conditions that have proven to be unfavourable...?)
   Practicing nurses:
   And vice versa, which working conditions make it more difficult for you to promote the participation of the users in their care?
   (Afterthought: Can you name further working conditions that have proven to be unfavorable...?)

11. *Key Informants:*
    Do the nurses who work here attend training or education or do they receive supervision that helps them to involve patients and relatives in their care? What are they and what do they contain?
    Practicing Nurses:
    Do or did you attend training or education, or do you receive supervision that helps you to involve patients and relatives in their care? If so, could you tell me more on what they contain?

Promotion of participation in groups and participation of communities as a responsibility of nurses

We are also interested in whether and how you work with patient/user groups and with communities.

12. *Key Informant interviews:*
    Do the nurses in your facility/health centre(s)/family health team(s) work with patient/user groups? Which groups exist and what are the tasks of the nurses there?
    Practicing nurses:
    Do you work with patient/user groups? Which groups are that and what are your tasks when you work with these groups?

a. *Key informants:*
   If question 12 "yes": In the work with patient/user groups, how do nurses strengthen the participation of the people in the group?
   In case of a possible 'misunderstanding' of IP: Let me better explicate the form of participation that I mean: How can nurses promote the active engagement of the people during the group meetings, for example that the group members share their experiences in the group)
   Practicing nurses:
   If question 12 "yes": In the work with patient groups, how do you strengthen the participation of the people in the group?
   In case of a possible 'misunderstanding' of IP: Let me better explicate the form of participation that I mean: How promote you the active engagement of the people during the group meetings, for example that the group members share their experiences in the group

b. *Key informants:*
Could you tell me about difficulties that nurses face when they would like to promote participation of patient/users during the group meetings?
Practicing nurses:
Could you tell me about difficulties that you face when you would like to promote participation of patients/users during the group meetings?
c. *Key informant Interviews: What supports nurses in their ability to work with groups? Can you tell me an example or a situation?*
Practicing nurses:
What supports you in your ability to work with groups? Can you tell me an example or a situation?

13. *Key informants:*
Do the nurse work in other community settings, e.g. in schools , youth centres, senior centres, and others? If so, in which way do they promote the participation of citizens in community health issues? (Could you give an example?)
Practicing Nurses:
Do you work in other community settings, e.g. in schools, youth centres, senior centres, and others? If so, in which way do you promote the participation of citizens in community health issues? (Could you give an example?)

14. *Key Informants:*
In your facility, health centre(s)/family health team(s), are there further projects or initiatives that have the objective to identify needs or problems of the community, where the nurses participate? Could you tell me more about that?
Practicing nurses:
Are you involved in further projects or initiatives that have the objective to identify needs or problems of the community? Could you tell me more about what you are doing?

15. *Key informants:*
In your opinion, which factors are supporting (could support) nurses' commitment in community participation, which factors are hindering it?
Practicing nurses:
In your opinion, which factors are supporting (could support) your commitment in community participation, which factors are hindering it?

Overall assessment on the relevance; facilitating and hindering conditions for strengthening user participation by nurses

16. *Key informants:*
Overall, how relevant is user participation for the nurses?
Practicing nurses:
Overall, how relevant is user participation in your daily work?
Key informants:
Overall, which are the most important conditions to strengthen the nurses to practically implement a greater participation of the users?
Practicing nurses:
Overall, which are the most important conditions that strengthen you in your daily work to practically implement a greater participation of the users?

17. *Key informant interviews:*
    And vice versa: Which conditions hinder the nurses to practically implement a greater participation of the users?
    Practicing nurses:
    And vice versa: Which conditions hinder you to practically implement a greater participation of the users?

    Thank you very much for your attention and for the interesting interview, I have asked all my questions. Is there something that you would like to add?
